# Distance between residence and the dialysis unit does not impact self-perceived outcomes in hemodialysis patients

**DOI:** 10.1186/1756-0500-5-458

**Published:** 2012-08-27

**Authors:** Paulo Roberto Santos, Francisco Plácido Nogueira Arcanjo

**Affiliations:** 1 Sobral School of Medicine, Federal University of Ceará, Avenida Comandante Maurcélio Rocha Ponte 100, Sobral, 62042-280, Brazil; 2 Avenida Comandante Maurocélio Rocha Ponte, 100 - CEP 62.042-280, Sobral, CE, Brazil; 3 Rua Tenente Amauri Pio, 380 Apt. 900 - CEP 60.160-090, Fortaleza, CE, Brazil

**Keywords:** Coping, Depression, End-stage renal disease, Hemodialysis, Quality of life, Distance effects

## Abstract

**Background:**

Patients have to travel long distances to undergo hemodialysis (HD) in some regions. We aimed to search for an association of the distance between patients’ residence and the dialysis unit with quality of life, depression and coping among end-stage renal disease (ESRD) patients undergoing maintenance HD.

**Methods:**

We studied 161 ESRD patients undergoing HD during April 2009. Quality of life, depression and coping were assessed by the SF-36, the 10-item CES-D and the Jalowiec Coping Scale, respectively. The sample was stratified in three groups: I-patients residing in Sobral (where the dialysis unit is located); II-patients residing in towns up to 100 km from Sobral; and III-patients residing in towns distant greater than 100 km from Sobral. Analysis of variance was used to detect differences in quality of life and coping scores between the groups. Logistic regression was used to test distance as a predictor of depression.

**Results:**

There were 47 (29.2%) patients residing in Sobral, 46 (28.6%) up to 100 km away and 68 (42.2%) greater than 100 km from Sobral. There were no differences related to quality of life and coping scores between the groups. Distance was not a predictor of depression.

**Conclusions:**

Social and cultural factors may explain the lack of differences. Studies from other regions are needed to clarify the distance effects on self-perceived outcomes among HD patients.

## Background

The most common modality of dialysis worldwide is hemodialysis (HD) [1]. In Brazil [2], 90% of end-stage renal disease (ESRD) patients undergo conventional HD performed in a hospital or satellite dialysis unit. Only 10% of ESRD patients are treated with some kind of home-based dialysis, mainly peritoneal dialysis and very rarely home HD [2]. The prevalence of ESRD patients on dialysis is lower in North and Northeast Brazil, respectively 279 and 357 per million, when compared to the 583 per million undergoing dialysis in the Southeast, the country’s most developed region [2]. There is no biological cause for the lower prevalence in the North and Northeast regions. Difficult access to dialysis treatment is the reason. One of the most common obstacles to accessing dialysis treatment is the inadequate number of dialysis units in large territorial areas, forcing patients to travel long distances to get specialized treatment. These long distances cause a substantial burden on patients.

The effects of long travel times on quality of life among HD patients have been demonstrated in a prospective observational study involving adult HD patients from the United States, Europe and Japan [3]. We have been studying quality of life [4-6] in a sample of patients treated with HD in a hospital dialysis unit that serves a population of 1,800,000 inhabitants spread over several small municipalities within a radius of 250 km (around 150 miles). Roughly, one-third of our patients live in the city where the dialysis unit is located, one-third live in cities located less than 100 km from dialysis unit and one-third in cities more than 100 km from the unit. Hence, we have a very suitable sample to study the effects of the distance between residence and dialysis unit on HD patients’ outcomes. Additionally, the lack of Brazilian data about these “distance effects” on HD patients’ outcomes makes our study more important.

Thus, we aimed to investigate whether there is an association of distance between patients’ residence and the dialysis unit with quality of life, depression and coping among ESRD patients undergoing maintenance HD.

## Methods

### Sample

The sample included ESRD patients undergoing HD during April 2009 in the only dialysis unit in northern Ceará state, northeastern Brazil. The criteria for inclusion were age older than 18, at least three months on dialysis and no previous transplantation. Out of 191 patients being treated by the unit that month, 161 were included. The reasons for exclusion were: 14 with less than three months on therapy, 8 with previous transplants, 5 refusals and 3 under 18 years old. All patients were undergoing conventional HD with polysulfone dialyzers (maximum number of reuses = 12). The study protocol and informed consent form were approved by the ethics committee of Vale do Acaraú University, which is the only ethics committee in our region.

### Measurement of quality of life

The measurement tool was the validated Brazilian version of the Medical Outcomes Study 36-Item Short Form Health Questionnaire (SF-36) [7]. The questionnaire was administered through interviews during April 2009, conducted by three professionals who did not belong to the dialysis unit team. This is a well-validated 36-item questionnaire covering issues relating to physical, psychological and social functioning. It generates scores from 0 (worst) to 100 (best) for eight sub-scales of quality of life: physical functioning, role-physical, bodily pain, general health, vitality, social functioning, role-emotional and mental health.

### Depression screening

For this analysis, the 10-item version of the Center for Epidemiologic Studies Depression Scale (CES-D) was used [8]. Respondents rate items by recalling the past week and using a three-point response scale, with higher scores indicating the presence and persistence of symptoms. A score ranging from 0 to 30 is calculated by summing the score of each item. A score ≥ 10 is classified as depression.

### Coping evaluation

The first version of the Jalowiec Coping Scale [9] was used, adapted to Portuguese by Souza-Talarico and colleagues [10], which consists of 40 items to determine coping style. This is a well-validated instrument, based on the cognitive theory of psychological stress and coping proposed by Lazarus and Folkman [11]. The 40 items are grouped into two styles: problem-oriented coping, comprising 15 items (scores ranging from 15 to 75), and emotion-oriented coping, comprising 25 items (scores ranging from 25 to 125). Problem-oriented coping aims to make direct changes in a stressful situation, whereas emotion-oriented coping seeks to ameliorate emotions associated with the problem. Subjects are evaluated about coping styles according to a five-point Likert scale, ranging from “never do” to “always do”.

### Patient data

The demographic data, time on dialysis and underlying etiology of ESRD were obtained from dialysis unit records. The underlying kidney disease was classified by clinical criteria and not by histopathology. Classification of socioeconomic status was according to criteria of the form issued by the Brazilian Association of Research Institutes [12]. This validated instrument is used in marketing surveys and population censuses and grades socioeconomic status into five subgroups: A (best status) through E (worst status). Besides income level, its criteria include educational level of the head of household and ownership of household appliances. Each patient was assigned a low, medium or high risk index based on comorbidity, as described by Khan et al. [13]. Khan’s comorbidity index takes into consideration age in three classes and nine comorbidities: diabetes, myocardial infarction, angina pectoris, congestive heart failure, liver cirrhosis, obstructive pulmonary disease, systemic collagen disease, pulmonary fibrosis and visceral malignancies. The laboratory results were those routinely measured in HD patients: creatinine and albumin (markers of nutritional status and inflammation activity), hemoglobin (level of anemia, current recommendation is partial anemia control, target = 11-12 g/dl), calcium and phosphorus (calcium and phosphorus product above 55 mg^2^/dl^2^ indicates risk of tissue deposition) and Kt/V (index of the dose of dialysis delivered, target ≥ 1.2). Kt/V was estimated using a second-generation Daugirdas formula [14].

### Statistical methods

We compared demographic, clinical and laboratory variables by stratifying the sample into three groups: I-patients residing in Sobral (where the dialysis unit is located); II-patients residing in towns up to 100 km from Sobral; and III-patients residing in towns distant greater than 100 km from Sobral. Comparisons of continuous and categorical variables were performed using the Kruskal-Wallis and Chi-square tests, respectively.

Logistic regression and analysis of variance were used for multivariate analysis. Logistic regression, adjusted for age, gender, time on dialysis, comorbidity, hemoglobin, albumin and Kt/V, was used to test the variable distance as a predictor of depression, with the category “living in Sobral” being considered as base and “up to 100 km from Sobral” and “greater than 100 km from Sobral” considered as risks. Analysis of variance, adjusted for age, gender, time on dialysis, comorbidity, hemoglobin, albumin and Kt/V, was used to detect differences of quality of life and coping scores according to the three established distance categories.

## Results

There were 47 (29.2%) patients living in Sobral, 46 (28.6%) up to 100 km away and 68 (42.2) greater than 100 km. There were no differences related to the sample characteristics according to the distance between the patients’ residence and the dialysis unit, as shown in Table 1. The distances of “up to 100 km” and “more than 100 km” between the patients’ residence and the dialysis unit were not predictors of depression (Table 2). Quality of life and coping scores also were not different according to the distance between the patients’ residence and the dialysis unit (Table 3 and Table 4).

**Table 1:** Comparison of sample characteristics according to the distance between the patients’ residence and the dialysis unit

**Variables**	**All sample**	**Living in Sobral**	**Distance ≤ 100 km**	**Distance > 100 km**	**P**
**Gender**					
Male	98 (60.9)	24 (51.1)	28 (60.9)	46 (67.6)	
Female	63 (39.1)	23 (48.9)	18 (39.1)	22 (32.4)	0.211
**Age**	44.5	46.1	44.5	43.4	0.794
**Economic class**^**a**^					
B	7 (4.3)	2 (4.3)	3 (6.5)	2 (2.9)	0.156
C	38 (23.6)	15 (31.9)	11 (23.9)	12 (17.6)	
D	94 (58.4)	28 (59.6)	26 (56.5)	40 (58.8)	
E	22 (13.7)	2 (4.3)	6 (13.0)	14 (20.6)	
**Etiology**					
Glomerulonephritis	69 (42.9)	19 (40.4)	20 (43.5)	30 (44.1)	0.919
Hypertension	38 (23.6)	9 (19.1)	11 (23.9)	18 (26.5)	0.670
Diabetes	16 (9.9)	9 (19.1)	3 (6.5)	4 (5.9)	0.056
Policystic kidney	11 (6.8)	1 (2.1)	5 (10.0)	5 (7.4)	0.233
Obstructive	7 (4.3)	3 (6.4)	1 (2.2)	3 (4.4)	0.642
Lupus	4 (2.5)	1 (2.1)	1 (2.2)	2 (2.9)	1.000
Indeterminate	16 (10)	5 (10.6)	5 (10.9)	6 (8.8)	1.000
**Time on dialysis** (months)	53.8	56.8	63.6	47.1	0.080
**Co morbidity**^**b**^					
Low	126 (78.3)	34 (72.3)	35 (76.1)	57 (83.8)	0.549
Medium	28 (17.4)	11 (23.4)	8 (17.4)	9 (13.2)	
High	7 (4.3)	2 (4.3)	3 (6.5)	2 (2.9)	
**Depression**					
Yes	13 (8.1)	6 (12.8)	2 (4.3)	5 (7.4)	0.330
No	148 (91.9)	41 (87.2)	44 (95.7)	63 (92.6)	
**Laboratory**					
Creatinine (mg/dl)	12.6	12.9	12.9	12.2	0.599
Hemoglobin (g/dl)	8.5	8.6	8.5	8.4	0.791
Albumin (g/dl)	4.3	4.3	4.3	4.3	0.807
**Calcium-phosphorus**					
product (mg^2^/dl^2^)	46.6	47.4	49.2	46.6	0.101
Kt/V	1.5	1.5	1.5	1.6	0.749

Data are means for continuous variables and percentages in parentheses.

^a^Brazilian Association of Research Institutes, B (best) and E (worst).

^b^Khan index.

**Table 2:** Logistic regression for depression according to the distance between patients’ residence and the dialysis unit

**Variable**	**≤ 100 km from dialysis unit**			**> 100 km from dialysis unit**		
	**OR**	**95% IC**	**P**	**OR**	**95% IC**	**P**
**Depression**	0.575	0.092-3.577	0.553	0.815	0.164-4.060	0.062

**Table 3:** Comparison of quality of life scores according to the distance between patients’ residence and the dialysis unit

**Quality of life dimensions**	**In the dialysis unit city**	**≤ 100 km from dialysis unit**	**> 100 km from dialysis unit**	**P**
**Physical functioning**	46.9	49.7	46.6	0.837
**Role-physical**	35.6	40.7	43.7	0.649
**Bodily pain**	49.9	54.2	61.5	0.172
**General health**	41.0	46.6	44.1	0.535
**Vitality**	50.0	48.2	52.2	0.720
**Social functioning**	65.6	67.3	65.5	0.959
**Role-emotional**	57.4	83.8	75.9	0.447
**Mental health**	58.2	55.2	59.1	0.737

Data are means.

**Table 4:** Comparison of coping scores according to the distance between patients’ residence and the dialysis unit

**Coping**	**In the dialysis unit city**	**≤ 100 km from dialysis unit**	**> 100 km from dialysis unit**	**P**
**P Coping**	52.4	50.3	51.3	0.743
**E Coping**	67.6	68.9	68.1	0.894

Data are means.

P Coping = Problem-oriented problem.

E Coping = Emotion-oriented problem.

## Discussion

As a pioneer Brazilian study, we tried to comprise broad self-perceived outcomes which were able to cover physical functioning and well-being (quality of life), feelings (depression) and behavior (coping).

Our hypothesis that patients who need to travel long distances to be treated with HD could have different self-perceived outcomes from patients living near the dialysis unit was not confirmed. The lack of similar studies in Brazil precludes comparisons. In the international literature, there are few studies about this question. The distance effects on death seem to be clearer [3,15].

In line with our results, a study comparing patients from in-center dialysis units with patients from satellite units found high prevalence of patients who need to travel more than 60 minutes among the in-center patients, but there were no differences between the groups regarding quality of life [16]. On the other hand, in the Dialysis Outcomes and Practice Patterns Study (DOPPS) [3], the SF-36 scores of patients spending more than 60 minutes traveling were lower than for those taking 15 minutes or less. Our patients living more than 100 km from Sobral spend much more than 60 minutes to get to the dialysis unit. Nevertheless, we did not find any difference related to their quality of life. When analyzing self-perceived issues, social and cultural factors can be involved. Our patients live in a low-income region where there are many municipalities with few resources. Thus people are used to traveling long distances to receive all kinds of services, including medical services. Perhaps because long travel time is routine among the patients, the distance effects do not exist”.

To our knowledge, our study is the first searching for distance effects on self-perceived outcomes among HD patients in Brazil. It is clear we need more studies, especially because Brazil has many regions where access to dialysis units is difficult. This difficulty is particularly severe in the North and Northeast regions, where dialysis units are concentrated in state capitals and there are vast territorial areas and few dialysis units in the countryside, creating distance barriers to patients’ access to treatment. Moreover, these regions are typically areas where home-based dialysis would be more suitable, like peritoneal dialysis and home HD, but these modalities are scarce. Home HD is not usual at all in any Brazilian region and peritoneal dialysis is seldom prescribed, mainly due to the lack of social and educational prerequisites. For our patients, the barriers to peritoneal dialysis are inadequate housing, difficulties due to appropriate family support and the association of lower education level with more peritonitis [17].

Since they come from a cross-sectional study, our results must be taken as preliminary. The lack of differences cannot indicate to health managers that HD patients living far from the dialysis unit present the same level of well-being as patients who do not travel to undergo HD. Health care givers in many regions of the globe have restructured dialysis unit geography with positive results [18-20]. We think Brazil is an excellent setting to study distance effects because of its characteristics: continental size, ideal number of dialysis units in developed areas and huge difficulties concerning accessibility to medical specialists in underdeveloped areas. And it must not be forgotten that scientific works can play an important role in encouraging an increase in the number and a more egalitarian distribution of dialysis units in Brazil. More studies on distance effects are urgent. We hope that this study will encourage other studies about accessibility and its effects on HD patients in Brazil.

## Conclusion

Our hypothesis that patients who need to travel long distances to be treated with HD could have different self-perceived outcomes from patients living near the dialysis unit was not confirmed. Social and cultural factors may explain the lack of differences. Studies from other regions are needed to clarify the distance effects on self-perceived outcomes among HD patients.

## Abbreviations

CES-D, Center for Epidemiologic Studies Depression Scale; ESRD, End-stage renal disease patients undergoing; DOPPS, The Dialysis Outcomes and Practice Patterns Study; HD, Hemodialysis; SF-36, The Medical Outcomes Study 36-Item Short Form Health Questionnaire.

## Competing interests

The authors declare that they have no competing interests.

## Authors’ contributions

PRS was responsible for the conception, design, analysis and interpretation of data. FPNA edited and made critical revision to the manuscript. Both authors read and approved the final manuscript.
